# Distinct impact modes of polygenic disposition to dyslexia in the adult brain

**DOI:** 10.1126/sciadv.adq2754

**Published:** 2024-12-18

**Authors:** Sourena Soheili-Nezhad, Dick Schijven, Rogier B. Mars, Simon E. Fisher, Clyde Francks

**Affiliations:** ^1^ Language and Genetics Department, Max Planck Institute for Psycholinguistics, Nijmegen, Netherlands.; ^2^ Donders Institute for Brain, Cognition and Behaviour, Radboud University, Nijmegen, Netherlands.; ^3^ Wellcome Centre for Integrative Neuroimaging, Centre for Functional MRI of the Brain (FMRIB), Nuffield Department of Clinical Neurosciences, John Radcliffe Hospital, University of Oxford, Oxford, UK.; ^4^ Department of Cognitive Neuroscience, Radboud University Medical Center, Nijmegen, Netherlands.

## Abstract

Dyslexia is a common and partially heritable condition that affects reading ability. In a study of up to 35,231 adults, we explored the structural brain correlates of genetic disposition to dyslexia. Individual dyslexia-disposing genetic variants showed distinct patterns of association with brain structure. Independent component analysis revealed various brain networks that each had their own genomic profiles related to dyslexia susceptibility. Circuits involved in motor coordination, vision, and language were implicated. Polygenic scores for eight traits genetically correlated with dyslexia, including cognitive, behavioral, and reading-related psychometric measures, showed partial similarities to dyslexia in terms of brain-wide associations. Notably, microstructure of the internal capsule was consistently implicated across all of these genetic dispositions, while lower volume of the motor cortex was more specifically associated with dyslexia genetic disposition alone. These findings reveal genetic and neurobiological features that may contribute to dyslexia and its associations with other traits at the population level.

## Teaser

Genetic disposition to dyslexia is associated with brain structure in the general population.

## INTRODUCTION

Roughly 3 to 7% of school-age children have dyslexia, a neurodevelopmental condition that affects reading, writing, and spelling (*1*). Reading acquisition during childhood is accompanied by the adaptation of several brain networks, and multiple hypotheses have been formulated to explain the etiology of dyslexia through altered developmental trajectories in these networks and the functions they support (*2*). The phonological deficit hypothesis suggests that dyslexia involves diminished ability in associating phonemes, the units of spoken language, with written linguistic symbols or graphemes, sometimes stemming from a lack of awareness of the phonological structure of language (*3*). In contrast, the orthographic deficit hypothesis suggests that some dyslexic readers may not identify words as cohesive patterns, but instead decode them as sequences of letters at a slow pace due to impairments of the visual stream (*4*). Yet, other mechanistic models highlight auditory (*5*) and magnocellular (*6*) pathways. Impairments of rapid automatized naming (*7*), verbal short-term memory (*8*), and attention control have also been implicated (*9*, *10*). Rather than there being a single, monolithic explanation for dyslexia, it is likely that the underlying mechanisms are heterogeneous and multifactorial (*11*, *12*).

Functional neuroimaging of people with dyslexia has suggested reduced activation or functional connectivity during reading-related tasks of various left-hemisphere regions that are important for language and/or normal reading, including the posterior temporoparietal cortex, the inferior frontal gyrus, and the anterior occipitotemporal cortex (*13*–*17*). However, these efforts often used divergent methods and task paradigms in sample sizes of only tens of individuals, and findings have often been inconsistent (*18*, *19*). In terms of brain structural magnetic resonance imaging (MRI), too, results from multicohort or meta-analysis studies in total sample sizes up to hundreds of individuals have not aligned well, yielding negative findings or much smaller effects than originally reported in smaller individual studies (*20*–*23*). Moreover, the largest diffusion MRI (dMRI)-based investigation of white matter microstructure, in 104 affected children and adolescents compared to 582 controls, did not detect significant groupwise differences (*24*).

This overall sequence has been encountered in neuroimaging studies of multiple other traits beyond dyslexia; initial waves of underpowered, hypothesis-driven studies produced inconsistent results, followed by larger more systematic screening studies that failed to replicate the initial findings, while sometimes producing unanticipated new leads (*25*). Together, it is clear that hypothesis-free brain-wide mapping in much larger sample sizes is needed, to better understand the brain regions and networks involved in dyslexia (*26*).

The heritability of dyslexia is estimated to be roughly 40 to 70% based on twin studies (*27*, *28*), with common DNA variants accounting for around 15% of its disposition according to genome-wide investigations (*29*, *30*). Dyslexia also shows substantial genetic correlations (in the range of 0.6 to 0.8) with measures of reading and spelling performance, and phonemic awareness, more broadly across the population (*31*). Here, we reasoned that estimating polygenic disposition to dyslexia in the UK Biobank, a large general population dataset where genome-wide genotype and neuroimaging data are available (*32*–*34*), would reveal neurobiological markers relevant to the development and/or manifestation of dyslexia. To calculate polygenic disposition in the UK Biobank individuals, we made use of genome-wide association summary statistics from a recent study of 51,800 individuals who reported having received a dyslexia diagnosis and over 1 million controls [using data from 23andMe Inc. (*30*)].

We carried out our brain mapping analyses with respect to voxel-wise volumetric measures, as well as fixel-wise apparent fiber density (AFD), the latter to study white matter microstructure. Different disposing genetic loci may affect distinct brain regions and networks. We therefore aimed to disentangle heterogeneity in the brain-wide associations of different dyslexia disposing variants, by decomposing the overall polygenic disposition into a number of distinct impact modes in terms of neurobiological correlates. For this, we developed an application of independent component analysis (*35*, *36*).

Dyslexia is also associated with several other traits related to cognition, education, and behavior and shows significant genetic correlations with attention-deficit hyperactivity disorder (ADHD), educational attainment, and intelligence (*30*). This means that some of the genetic factors that dispose to dyslexia are shared with these other traits. The question then arises: Which structural brain features are associated with polygenic disposition to dyslexia alone, versus more generally with polygenic dispositions to a range of cognitive, educational, and behavioral traits that are associated with dyslexia? The combination of brain features uniquely associated with dyslexia polygenic disposition is likely to distinguish liability to this particular trait among others. We therefore went on to quantify the polygenic dispositions of UK Biobank individuals to ADHD, educational attainment, school grades, fluid intelligence, and the reading-related psychometric traits of single-word reading, nonword reading, spelling, and phonemic awareness (*31*). We mapped the brain structural correlates of all of these polygenic dispositions in the UK Biobank and compared and contrasted with the brain maps for dyslexia polygenic disposition.

## RESULTS

### Brain correlates of dyslexia polygenic scores

After genetic and brain imaging quality control, we generated dyslexia polygenic scores (PGSs) for between 31,695 and 35,231 adult individuals from the UK Biobank dataset, depending on the availability of data for diffusion and T1-weighted MRI modalities, respectively (see Materials and Methods). We mapped brain wide associations of dyslexia PGS with voxel-wise regional volume derived from tensor-based morphometry (*37*, *38*), as well as microstructural measure of AFD derived from fixel-based analysis (*39*) (Materials and Methods). For our main analysis, we report results for PGS generated with Lassosum2 (*40*) that were optimized for capturing inter-individual brain variation (Materials and Methods; fig. S1), but other automated polygenic methods including SBayesR (*41*) and PRS-CS (*42*) delivered highly comparable results (fig. S2).

**Fig. 1.**
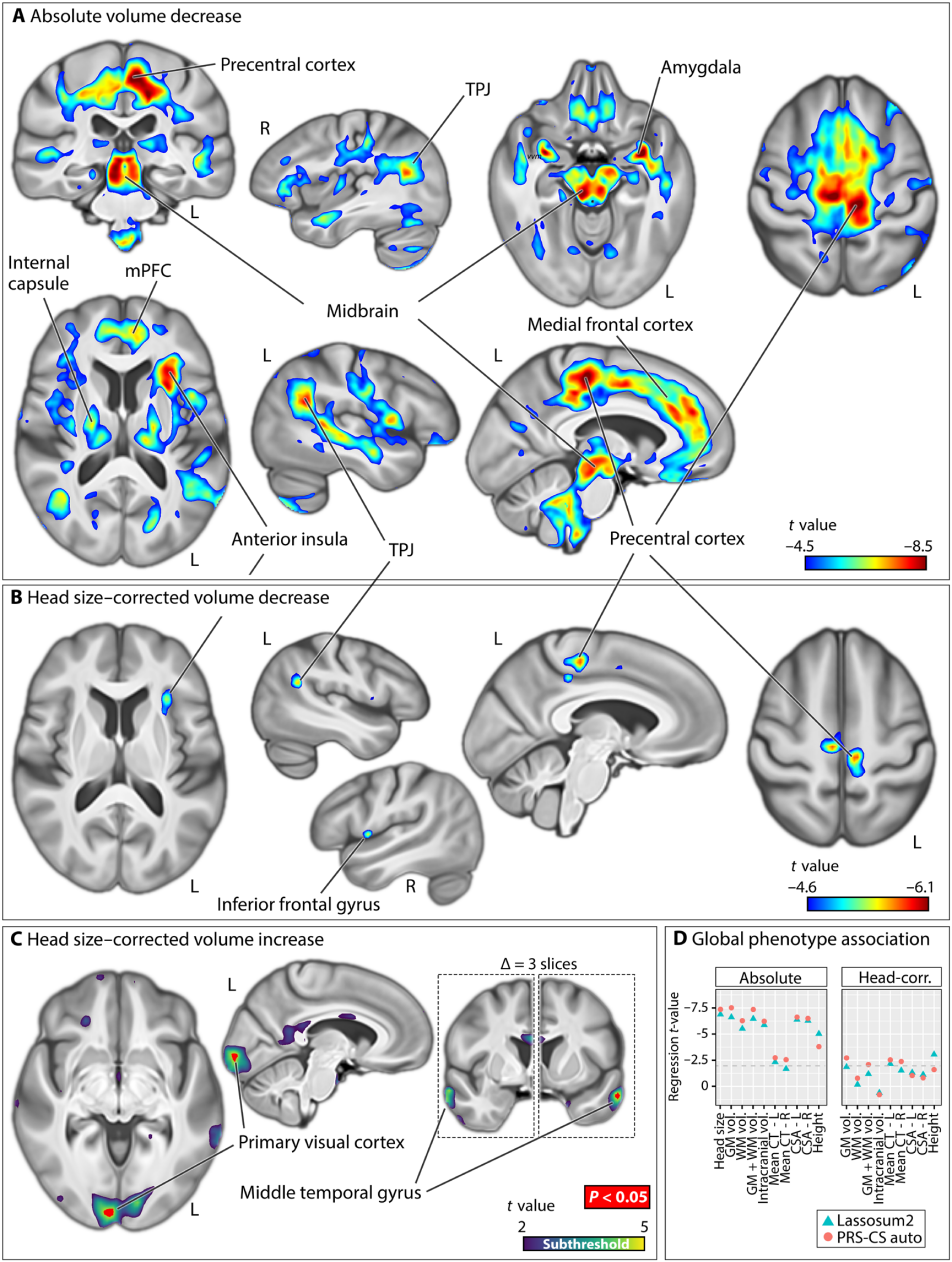
Associations of dyslexia PGSs with regional brain volume. Negative dyslexia PGS associations with regional brain volume before (**A**) and after head size correction (**B**). Clusters in (A) and (B) indicate voxels whose volumes are significantly lower in individuals with higher polygenic disposition to dyslexia, at *P* values of smaller than 0.05 as obtained from nonparametric testing, with brain-wide correction for multiple comparisons using 5000 permutations. In these significant clusters (rainbow), voxels are colored based on *t* values derived from similar parametric tests, to visualize effect sizes and peak regions. In (**C**), positive associations in the head-size–corrected model are depicted (where higher dyslexia PGS is associated with increased regional volume relative to head size: Viridis colors indicate subthreshold (i.e., nonsignificant) clusters and red depicts voxels passing brain-wide significance). (D) shows the associations of dyslexia PGS with various global brain metrics and individuals’ heights. Figures are shown in radiological convention, where the left side in transverse and coronal views corresponds to the right cerebral hemisphere and vice versa. R, right; L, left; TPJ, temporoparietal junction; CT, cortical thickness; GM, gray matter; WM, white matter; Vol, volume; CSA, cortical surface area. Lasso-sum and PRS-CSauto refer to different methods for calculating polygenic scores (see Materials and Methods).

Individuals with higher dyslexia PGS exhibited lower total brain volume, which was more apparent in gray matter than white matter (*t* = −6.6 and *t* = −5.5, respectively; Fig. 1D). Among other global measures of brain anatomy, dyslexia PGS was most strongly associated with lower total cortical surface area, especially of the left hemisphere (*t* = −6.4). Unexpectedly, dyslexia PGS was slightly more predictive of overall head size (*t* = −6.9, *r^2^* = 0.14%, and *P* < 10^−11^), which is a measure derived from skull anatomy (*43*), than any brain measure (Fig. 1D). Head size or total brain volume is commonly used as covariates in structural neuroimaging analysis, when trying to map region-specific effects that are independent of global effects. However, head size and brain volume are partly heritable traits, so that treating them as confounding variables in genetic association analysis may sometimes have unintended consequences for assessing the presence and directions of associations, a phenomenon described in detail by Aschard *et al.* (*44*). Therefore, we performed our primary voxel-wise analysis without this adjustment (i.e., using raw Jacobian determinant values comprising both the nonlinear and linear registration components), but also repeated the analysis secondarily with head size treated as a confounding covariate.

Without head size adjustment, individuals with higher dyslexia PGS showed lower regional volumes across multiple brain regions after brain-wide multiple comparison correction (Materials and Methods). This included a large frontal cluster along the medial wall, extending from Brodmann area 4 to perigenual medial frontal cortex (Fig. 1A). In addition, lower volume was observed in midbrain, thalamus, and bilateral amygdalae, in individuals with higher dyslexia PGS (Fig. 1A). Many of these associations were evident in both hemispheres and more or less bilaterally symmetric, except two clusters of lateralized lower volume in the left anterior insula and in the left posterior temporoparietal junction, again associated with higher dyslexia PGS (Fig. 1A). There were no regions where higher dyslexia PGS was significantly associated with higher (rather than lower) regional volumes in voxel-wise analysis after multiple comparison correction. A post hoc regression analysis using log-transformed Jacobian determinant values (instead of raw values) yielded an effectively identical brain-wide *t*-map as the original analysis without this transformation (Pearson’s *r* ≅ 1.0; maximum absolute voxel-wise *t* value = 8.44 in the log-transformed model, compared to 8.48 in the original model.)

Following adjustment for head size as a confounding covariate, negative associations were again observed between dyslexia PGS and similar brain regions as the nonadjusted analysis, although the significant clusters were markedly smaller compared to the nonadjusted analysis (Fig. 1B). In addition, some significant positive voxel-wise associations emerged after head size correction, in the primary visual cortex and anterior middle temporal gyrus, indicating that individuals with higher dyslexia PGS had higher volumes relative to their head size in these regions (Fig. 1C).

**Fig. 2.**
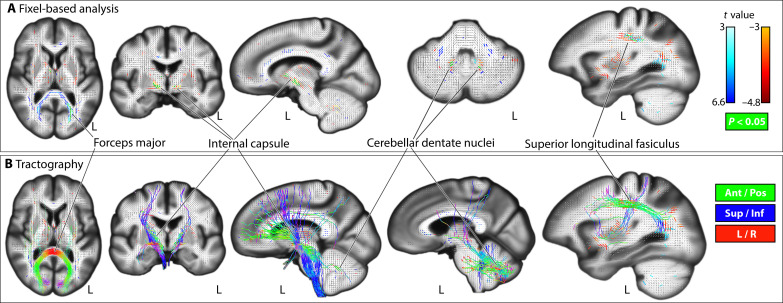
Associations of dyslexia PGSs with white matter microstructure. Association of dyslexia PGS with fixel-wise apparent fiber density (**A**). Probabilistic tractography from significant fixels [*P* < 0.05, 5000 permutations; (**B**) streamlines in B are colored based on fiber directions] Figures are shown in radiological convention, where the left side in transverse and coronal views corresponds to the right cerebral hemisphere and vice versa. Ant, anterior; Pos, posterior; Sup, superior; Inf, inferior.

In fixel-wise analysis of white matter microstructure, dyslexia PGS was positively associated with AFD in forceps major tracts, which connect homologous regions of the bilateral occipital cortices (Fig. 2A). In contrast, dyslexia PGS was negatively associated with AFD in three separate clusters of fixels bilaterally: within the superior longitudinal fasciculi, cerebellar dentate nuclei, and anterior limb of the internal capsule (Fig. 2A). Fiber tractography revealed that the internal capsule and dentate fixels highlight tracts that pass through the brainstem and superior cerebellar peduncles, with neocortical connections that mainly span the frontal and parietal cortices (Fig. 2B). The global mean AFD per participant was not correlated with dyslexia PGS (*r^2^* = 1.4 × 10^−5^ and *P* > 0.05), such that adjusting for this metric as a covariate in post hoc analysis yielded an almost identical brain-wide association map as the primary, unadjusted analysis (*r* = 0.98, with a maximum absolute fixel-wise *t* value of 6.59 for the adjusted model, compared to 6.61 in the primary model).

### Heterogeneous brain-wide associations of dyslexia disposing genetic loci

**Fig. 3.**
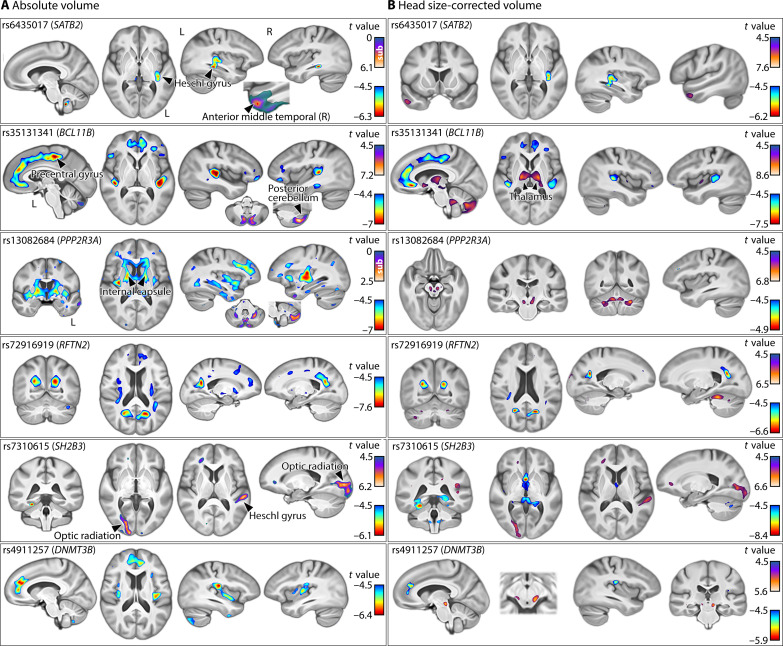
Associations of single dyslexia-disposing genetic variants with brain volume. Voxel-wise associations with six variants that disposed to dyslexia at a genome-wide significant level and exhibited volumetric associations across regions surpassing 5 cm^3^ in total. Panels show associations with regional brain volume unadjusted (**A**) and adjusted (**B**) for head size. Rainbow clusters highlight the associations of dyslexia-disposing alleles with lower regional volume, and inferno clusters highlight opposite associations. Brain maps of all 35 genome-wide significant variants are also provided in data S1.

There were 42 independent, genome-wide significant loci associated with dyslexia in the 23andMe Inc. genome-wide association study (GWAS) (*30*). For 35 of these loci the lead genetic variant in the UK Biobank data passed our quality filtering (Materials and Methods). We mapped the brain-wide associations for each of these 35 genetic variants separately, with reference to increased dosage of the disposing alleles. The 35 brain-wide maps showed some limited convergence, most notably in a left hemisphere medial prefrontal region peaking in Brodmann area 32 (*45*) that was associated with six of the variants, but there was also much divergence across the 35 maps (Fig. 3 and data S1).

For example, for voxel-wise volumetric analysis, the locus intronic to *PPP2R3A* [index single-nucleotide polymorphism (SNP) rs13082684, which was the most significant dyslexia-disposing variant in the 23andME GWAS] was associated with lower volume in the right posterior insula and Heschl’s gyrus, and deep subcortical structures spanning the internal capsule, anterior thalamus, and anterior thalamic radiations (Fig. 3). The intronic *BCL11B* variant (rs35131341) was associated with lower volume in medial frontoparietal areas, bilateral Heschl’s gyri, and planum temporale (Fig. 3). In contrast, association in the opposite direction (i.e., increased volume with the disposing allele) was observed in posterior cerebellum for the *BCL11B* variant (Fig. 3). The *SATB2* locus (rs6435017) exhibited lateralized association with lower volume of the posterior insula and left Heschl gyrus, and higher volume in the right anterior middle temporal gyrus (Fig. 3). The *AUTS2* variant (rs3735260) was associated with higher volume in the optic radiation close to the primary visual cortex, with a more pronounced effect in the right hemisphere, while the *SH2B3* locus (rs7310615) was also associated with higher volume in the right optic radiation, as well as increased volume in the left Heschl’s gyrus (Fig. 3). Brain-wide volumetric association maps for all 35 genome-wide significant dyslexia-disposing variants are in data S1.

After adjusting for head size as a covariate, the brain-wide association maps remained similar for most of these 35 variants, although the *PPP2R3A* dyslexia-disposing variant was now associated with higher volumes in the cerebellum and midbrain rather than widespread regional volume reductions, and the association with the *BCL11B* variant extended to the thalamus (Fig. 3 and data S1).

In terms of white matter microstructure, again the 35 genetic variants had mostly distinct brain-wide association maps (fig. S3). For example, a variant upstream of *NEUROD2* (rs12453682) was associated with lower AFD in tracts passing through the internal capsule and caudally extending to the brainstem and superior cerebellar peduncles (fig. S3). Other negative associations were observed in the superior longitudinal fasiculi for the *SH2B3* (rs7310615) and *SEMA3F* (rs2624839) loci (fig. S3). In contrast, positive associations were observed in the occipital lobes for the *SEMA3F* (rs2624839) and *ARFGEF2* (rs11393101) loci, such that higher AFD in the forceps major tracts was associated with the dyslexia-disposing alleles (fig. S3). Fixel-wise associations for all 35 genome-wide significant dyslexia-associated variants are in data S1. Adjusting for global mean AFD as an additional covariate made little difference to these association maps, with correlations between fixel-wise values before and after adjustment ranging from 0.92 to 1.0 across the 35 variants (data S1).

### Latent structure in imaging genetic associations revealed by impact modes

As described in the previous section, 35 genetic variants that were individually associated with dyslexia at a genome-wide significant level had largely distinct, but sometimes overlapping, associations with regional brain volumes or white matter microstructure. We opted to broaden this insight to thousands of genetic variants that contribute to the polygenic disposition to dyslexia, through an application of probabilistic independent component analysis (*36*). We aimed to understand whether, despite heterogeneity of brain-wide associations for different genetic variants, there exists a latent multivariate structure. This approach goes beyond the standard PGS approach that aggregates all disposing variants into a single scalar score per subject.

We first mapped the brain-wide associations in the UK Biobank data for each of 13,766 genetic variants that were associated with dyslexia in the 23andMe GWAS (*30*) with pointwise association *P* values of less than 0.01 and clumped for linkage disequilibrium (Materials and Methods). Together, these variants contribute much of the genome-wide polygenic disposition to dyslexia. We then concatenated the resulting 13,766 brain-wide association maps and decomposed them into 10 independent components, separately for voxel- and fixel-wise data (see Materials and Methods). Each component reflects a spatially independent map of brain regions associated with a distinctive set of genetic variants that exhibit similar brain-wide effects. We call these components impact modes, a term we partly borrowed from Smith *et al.* (*46*).

**Fig. 4.**
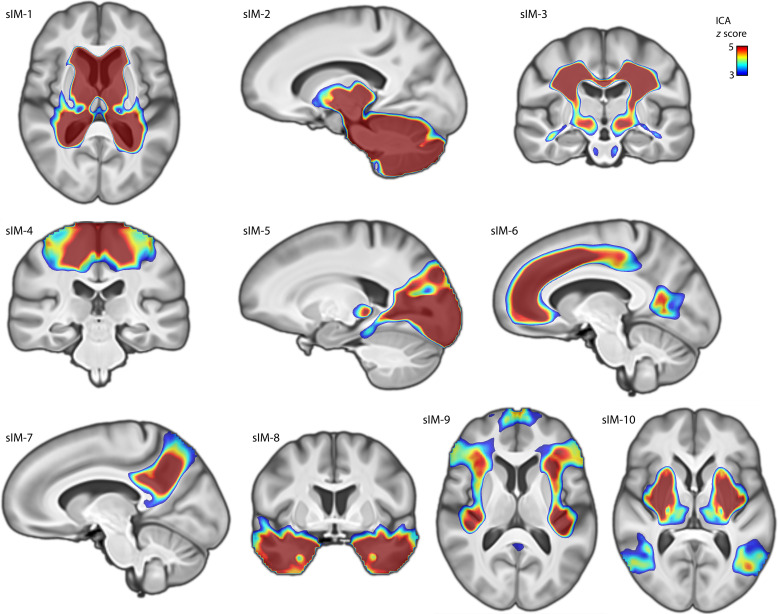
Dyslexia genomic-brain-sIMs. Ten impact modes identified by independent component analysis of brain morphometry *t* maps corresponding to the top 13,766 independent dyslexia-disposing variants that contribute to polygenic disposition. *Z* scores indicate the contribution and “weight” of each voxel in the corresponding independent component. Through this analysis, the overall polygenic disposition to dyslexia was decomposed into distinct spatial components in terms of contributing genetic variants and their specific brain-wide associations. sIM, structural impact mode.

We found that 10 impact modes explained totals of 25.5 and 8.5% of variance in brain-wide *t* maps, respectively in voxel- and fixel-wise data. Impact modes localized to anatomically coherent features and were more spatially homogenous than the univariate brain maps associated with dyslexia PGS (Fig. 4). For example, in the voxel-based volumetric data, impact mode #5 mapped distinctly to the occipital lobes and posterior thalami [Fig. 4: structural impact mode (sIM) #5]. Among genetic variants that contributed especially to this impact mode, a dyslexia-disposing variant at the *DAAM1* locus was associated with increased volume of the primary visual cortex (rs36065072, mode weight *z* score = 6.2). A further example to illustrate: Impact mode #8 exclusively captured bilateral associations with temporal lobes (Fig. 4: sIM #8), and an intronic variant in *MAPT* was among those that exhibited a strong weight in this impact mode (rs12150530, mode weight *z* score = −4.7). The weights of all 13,766 variants for the 10 sIMs are provided in table S1.

**Fig. 5.**
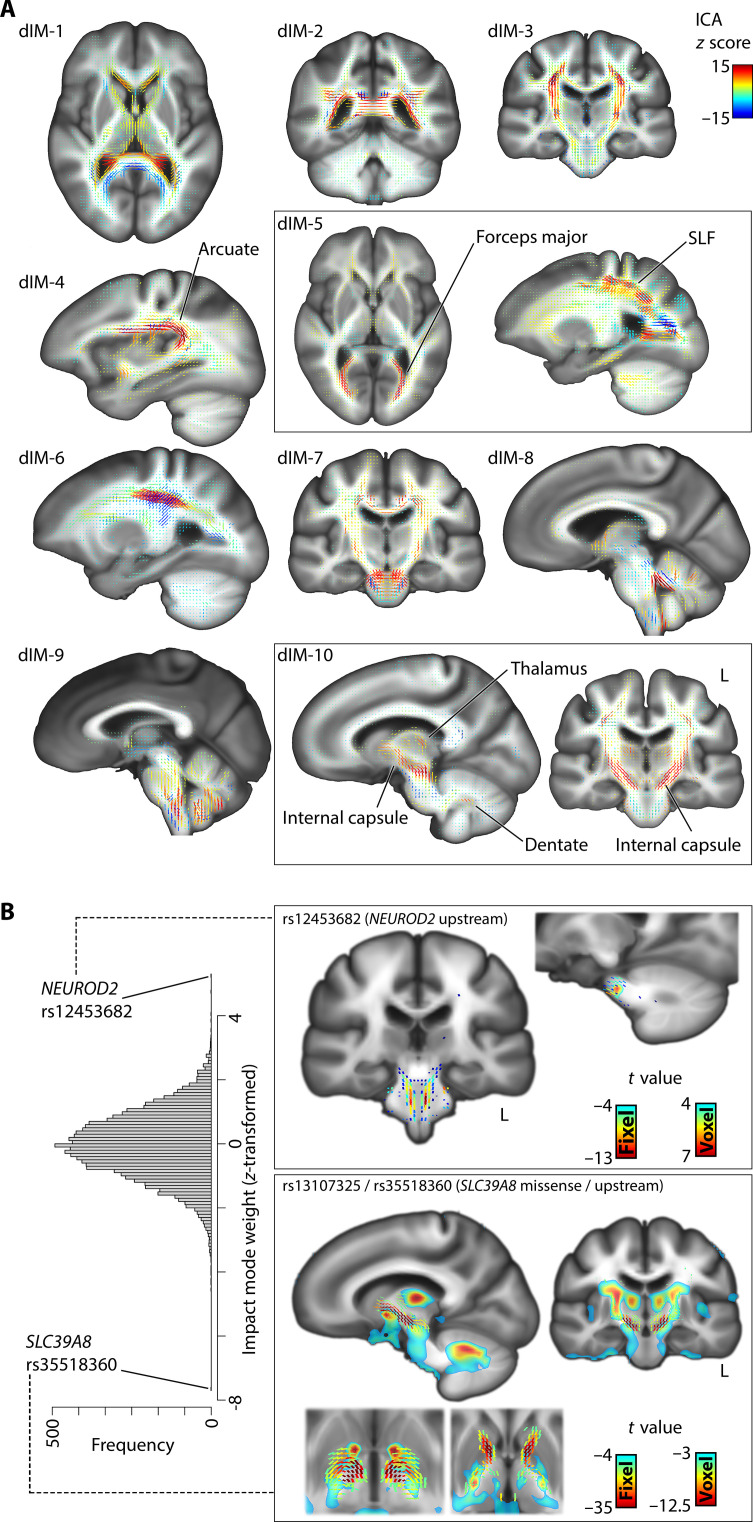
Dyslexia diffusion impact modes. (**A**) Distinct impact modes identified by independent component analysis (ICA) of fixel-wise associations, for 13,766 variants that contribute to genome-wide polygenic disposition to dyslexia. (**B**) Histogram of variant-wise weights for impact mode #10, and univariate maps of the two top loci contributing to this impact mode, *NEUROD2* and *SLC39A8*, as examples of how an impact mode can be queried in terms of specific genetic contributions. SLF, superior longitudinal fasciculus.

Impact modes for fixel-based white matter microstructure corresponded to groups of identifiable tracts. For example, diffusion impact mode #5 captured microstructural variations in the forceps major, optic radiation, and superior longitudinal fasciculus, whereas impact mode #10 isolated the internal capsule and brainstem tracts that caudally enter superior cerebellar peduncles (Fig. 5A). While both of these sets of tracts were associated with the overall dyslexia PGS (Fig. 2), their association with two independent modes indicates that they stem from distinct genomic underpinnings. The genetic variant with the strongest weight for the internal capsule impact mode (#10) was upstream of *SLC39A8* (rs35518360, mode weight *z* score = −7.6) (Fig. 5B). This variant was only weakly associated with dyslexia (GWAS, *P* = 0.001) but has also been significantly associated with schizophrenia (*47*) and educational attainment (*48*). The variant is in almost full linkage disequilibrium (*r*^2^ = 0.9) with a missense variant (rs13107325) in the same *SLC39A8* gene, suggesting that an amino acid change in the encoded protein, which is a divalent cation transporter, affects microstructural properties of the internal capsule tracts. The dyslexia-disposing *NEUROD2* variant (rs12453682) exhibited the second-strongest weight for this same impact mode (#10) (mode weight *z* score = 5.3) (Fig. 5B). The complete weights of the 13,766 genetic variants for all 10 diffusion impact modes are provided in table S2.

### Overlap in brain-wide associations of dyslexia PGSs and genetically correlated traits

Consistent with previous reports (*30*, *31*), using GWAS summary statistics from large-scale genetic studies of other cognitive, educational, and behavioral traits (see Materials and Methods), we reproduced significant SNP-based genetic correlations between dyslexia and GCSE education (General Certificate of Secondary Education in the UK) (*r*_g_ = −0.50), verbal-numerical reasoning (*r*_g_ = −0.49), the first principal component of school grades (*r*_g_ = −0.39), ADHD (*r*_g_ = 0.40), word reading ability (*r*_g_ = −0.69), nonword reading ability (*r*_g_ = −0.71), spelling (*r*_g_ = −0.75), and phonemic awareness (*r*_g_ = −0.62) (all with *P* values < 10^−23^) (fig. S4).

**Fig. 6.**
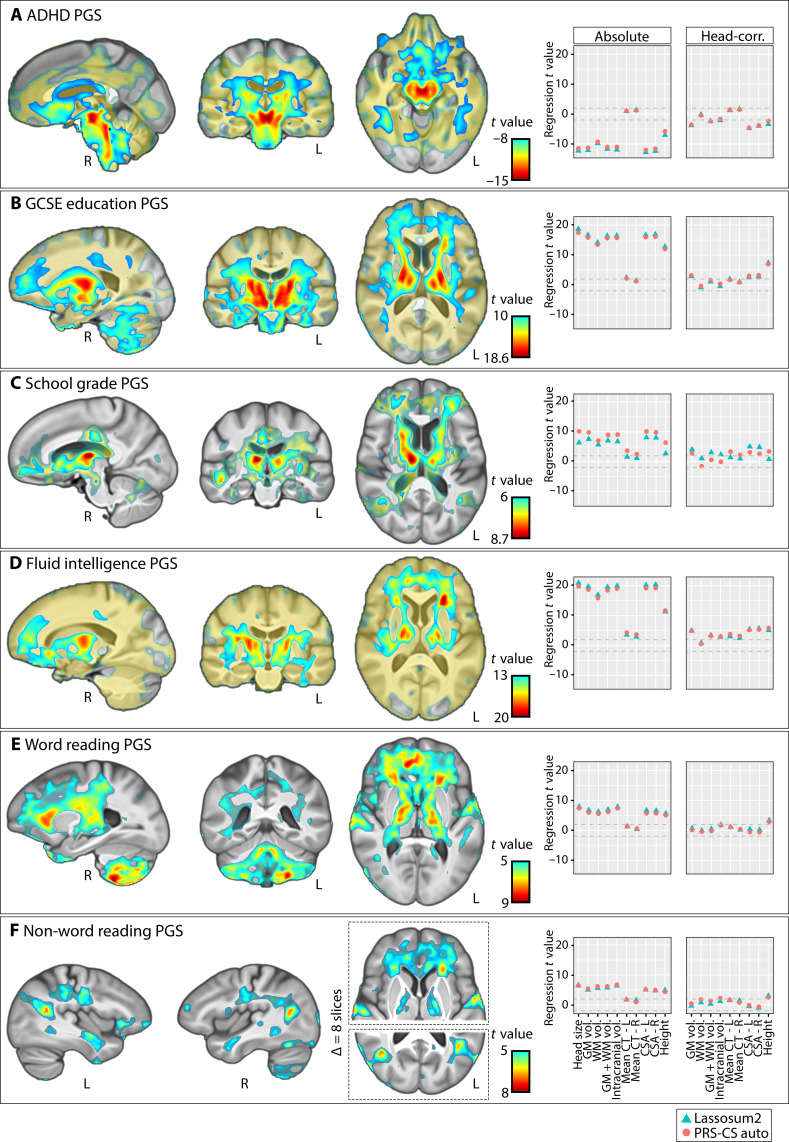
Brain-wide volumetric associations of PGSs for traits that are genetically correlated with dyslexia. [(**A**) to (**F**)] The panel on the left shows the associations of PGS with regional brain volumes. The panel on the right shows the associations of PGS with global imaging measures, before and after adjustment for head size as a covariate (dashed lines indicate a *t* value of 2). PGS for phonemic awareness was not significantly associated with the volume of any voxel and therefore not shown in the figure. The PGS for spelling ability only showed a significant association in the putamen and is also not shown in this figure (its map is shown in fig. S5). IDP, imaging-derived phenotype.

We then generated PGS for each of these traits in the UK Biobank imaging genetic dataset using Lassosum2 (*40*) (i.e., the same method as for our primary analysis of dyslexia PGS above). PGSs for several of these traits were associated with the volumes of basal ganglia, thalamus, and adjacent white matter tracts, especially in the internal capsule (Fig. 6). These associations extended to the frontal lobes for the fluid intelligence PGS, education PGS, and school grade PGS and more caudally to the brainstem for ADHD PGS. The directions of effects were consistent across traits, with polygenic disposition to poorer performance, lower education, and ADHD associated with lower regional volumes in these areas (Fig. 6), similarly to dyslexia PGS (Fig. 1).

A lateralized association between higher dyslexia PGS and lower left anterior insula volume (Fig. 1) was also observed for PGSs for lower fluid intelligence, word reading, nonword reading, and GCSE education (Fig. 6). Lower temporoparietal junction volume, which was observed in individuals with higher dyslexia PGS (Fig. 1), was also observed in individuals with lower nonword reading PGS (Fig. 6). In contrast, as a feature that was only found for dyslexia PGS, the association with Brodmann area 4 (primary motor cortex) (Fig. 1) was not observed as a focus for PGS of other traits (Fig. 6). PGSs for word reading and nonword reading were positively associated with the bilateral volumes of Heschl’s gyri that contribute to primary auditory cortex, as well as posterior cerebellum (Fig. 6). No associations were observed between PGSs for lower performance in any trait and higher regional brain volume, with the exception of spelling, for which individuals with polygenic disposition to lower performance showed higher volume in the putamen (fig. S5).

**Fig. 7.**
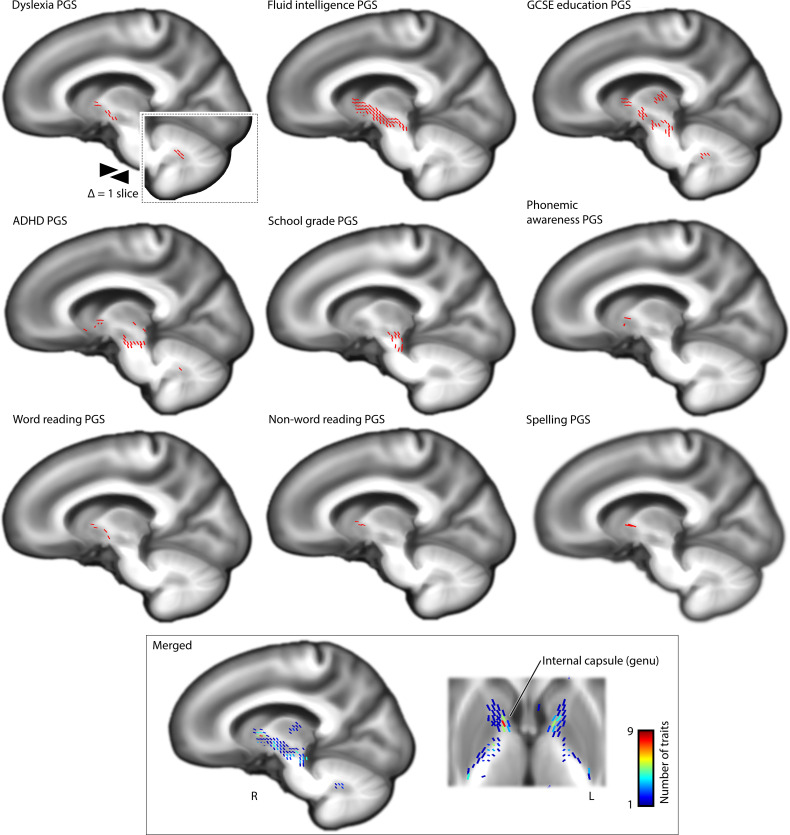
White matter microstructural associations of PGSs for traits that are genetically correlated with dyslexia. PGSs of additional traits that are genetically correlated with dyslexia show associations with white matter microstructure, measured by apparent fiber density. Lower apparent fiber density in the internal capsule was consistently associated with polygenic disposition to lower performance/achievement and higher risk for ADHD. The genu of the internal capsule was a hotspot of shared association with PGSs for all traits.

In white matter fixel-based analysis, associations were observed between AFD in the internal capsule tracts and PGSs for all traits (Fig. 7). Specifically, lower AFD was consistently associated here with polygenic disposition to lower performance/achievement and higher risk for ADHD. PGS for fluid intelligence and GCSE education exhibited the most extensive associations here, passing through the anterior limbs of the internal capsule (Fig. 7). In particular, the genu and anterior limb of the internal capsule emerged as a hotspot, where PGS for dyslexia and all other genetically correlated traits overlapped in their associations (Fig. 7). In addition, lower PGS for GCSE education and higher PGS for ADHD were associated with lower AFD in cerebellar dentate nuclei, similarly as for higher dyslexia PGS.

We found that dyslexia PGS was weakly but significantly correlated with the phenotypic traits of fluid intelligence (*r* = −0.11, *t* = −21.7, and *P* < 10^−16^) and years of education (*r* = −0.047, *t* = −8.8, and *P* < 10^−16^). After including these two traits as additional covariates, all clusters in voxel-wise volumetric analysis remained significantly associated with dyslexia PGS, apart from the left anterior insula that was no longer significant (fig. S6). For white matter, all fixel clusters remained significant after adjustment for fluid intelligence and years of education, namely, in the internal capsule, forceps major, and superior longitudinal fasciculus (data S1).

## DISCUSSION

Our study of genetic disposition to dyslexia in over 30,000 adults implicated diverse brain structures, notably involved in motor, language-related, and visual functions. Our sample size was more than two orders of magnitude larger than any neuroimaging case-control study of dyslexia published to date, which is likely to have aided in robustness of our findings. Nonetheless, a direct comparison cannot be made to case-control studies. Rather, our study shows the utility of a complementary approach to studying the neurobiology of dyslexia, through identifying neural correlates of genetic disposition while leveraging large-scale population data to overcome statistical uncertainty.

Independent component analysis identified various impact modes comprising sets of dyslexia-disposing genetic loci associated with distinct brain features. This heterogeneity is consistent with dyslexia as a high-level behavioral outcome, with no simple mapping to any single brain structure, network, cognitive function, or genetic factor. Dyslexia emerges from a complex interplay between genes, environmental exposures, and neural adaptations during reading acquisition (*2*, *11*, *12*, *28*) and is associated with educational and socioeconomic outcomes (*49*, *50*). Some of the structural brain correlates of polygenic disposition in the adult population may therefore be linked with the development of dyslexia as potential causal factors, while others might be consequences of lifestyle differences, for example, time spent reading professionally or personally.

Several of the implicated brain structures were also associated with genetic dispositions to other traits including educational attainment, fluid intelligence, ADHD, and reading-, and language-related performance measures across the population. This is consistent with dyslexia at the population level showing substantial phenotypic and genetic correlations with these other traits, rather than having an entirely distinct etiology of its own, i.e., many genomic variants that dispose to dyslexia are likely to be pleiotropic for these other traits. However, the volume of a large continuous region along the medial wall, spanning parietal and frontal cortices and peaking within the primary motor cortex (*51*), showed an association only with genetic disposition to dyslexia among all of these traits. A combination of reduced primary motor cortex volume together with alterations of other regions implicated by this study may therefore dispose individuals to dyslexia in particular. Perhaps consistent with this, children with dyslexia have shown overactivation of the primary motor cortex during reading or reading-related tasks (*52*). Furthermore, at the phenotypic level, dyslexia is associated with motor difficulties (*53*, *54*), although many children with dyslexia show no motor impairments, and lower performance of sequential motor tasks has also been reported for ADHD (*53*, *54*).

Lower volume of the medial wall region spanning the primary motor cortex was notably associated with the dyslexia-disposing allele of the *BCL11B* variant. This allele was also associated with higher volume of the posterior cerebellum. Consistent with this, *BCL11B* encodes a zinc finger protein transcription factor and is expressed in cerebral cortical layer V projection neurons that send motor connections to the brainstem and cerebellum (*55*–*58*). *BCL11B* may therefore modulate the topology of corticocerebellar pathways. Rare missense variants of *BCL11B* are associated with speech impairment, developmental delay, and intellectual disability (*59*).

Of further relevance in terms of motor circuits, a consistent finding across all of the PGSs that we studied, including dyslexia PGS, involved microstructure of the internal capsule. A clue to the role of this deep white matter tract in dyslexia is provided by diffusion impact mode #10, which isolated the internal capsule and the cerebellar dentate nuclei together as one single component, linked to a shared set of dyslexia-disposing genetic variants. Motor projections such as the dentate-thalamic tracts pass through the internal capsule, while dentate nucleus lesions are the cause of cerebellar cognitive affective syndrome that involves linguistic, executive, and visual-spatial impairments (*60*). Especially, the internal capsule’s anterior limb was the main focus of convergence across the various PGS that we studied. This region reciprocally connects the thalamus and frontal cortex and is engaged in multiple cognitive domains that contribute to psychiatric traits (*61*).

Specific genetic loci that contributed strongly to the internal capsule and dentate impact mode #10 included the *SLC39A8* and *NEUROD2* loci. *SLC39A8* encodes a metal ion transporter that modulates neurotransmitter receptor glycosylation (*62*), and this locus has also shown genome-wide significant associations with schizophrenia (*47*), intelligence (*63*) educational attainment (*48*), and quantitative magnetic susceptibility—an MRI-based measure that is sensitive to iron deposition, myelin, and calcification—in the caudate, substantia nigra, and pallidum (*64*). *NEUROD2* codes for a neuronal migration and differentiation factor and its expression colocalizes with *BCL11B+* layer V pyramidal neurons (*65*). In mice, *NEUROD2* knockout selectively increases excitability of layer V neurons (*65*), while in humans, haploinsufficiency of *NEUROD2* is associated with intellectual disability, autism, and speech delay (*65*). Together with our findings across PGS for dyslexia and various other genetically correlated traits, the internal capsule and cerebellar dentate nucleus may be involved in these traits through altered corticocerebellar circuits that require layer V projection neurons, with consequences for diverse aspects of cognition, including those required for normal reading.

In line with the implication of left-lateralized language-related brain regions by some previous studies of dyslexia (see Introduction), we found that higher dyslexia PGS was associated with lower regional volume in the left temporoparietal junction and left anterior insula. The left temporoparietal junction is involved in the processing of syntactic and semantic domains of language and coding and retrieving speech sounds (*66*–*68*). The PGSs for nonword reading and school grades were also associated with temporoparietal junction volume, further supporting the relevance of this brain region for phonological decoding ability and educational outcomes. Regarding the anterior insula, this region is closely connected to the adjacent inferior frontal gyrus (*69*, *70*), another core region that participates in the language network. Insular activation recently emerged as a focus of convergence in a meta-analysis of functional MRI studies using rapid naming, rapid word reading, and rapid sentence reading tasks (*71*). The left anterior insula was also associated with the fluid intelligence PGS in our study. This region may therefore contribute to dyslexia in terms of both rapid reading fluency and/or more generally through higher cognitive processes. Consistent with this, when we adjusted for the phenotypic traits of years of education and fluid intelligence in post hoc analysis, the association of dyslexia PGS with the left anterior insula volume was no longer significant, while the other regional associations remained.

We found that several of the individual genetic loci that dispose most significantly to dyslexia were associated with the volumes of primary auditory cortices (Heschl’s gyri), including *PPP2R3A, BCL11B, SATB2,* and *SH2B3*. This supports an involvement of primary auditory cortex in the neural basis of dyslexia, as has been discussed previously (*72*). However, the effects that we observed were different across the genetic loci in terms of directions of effect (volumetric increases or decreases) associated with the dyslexia disposing alleles, even within the same hemisphere. This pattern might arise because altered Heschl’s gyrus volumes may be only secondary to the molecular and cellular roles of these genes in auditory cortex function that are relevant for dyslexia.

Regarding visual circuits, increased polygenic disposition to dyslexia was associated with increased AFD in the forceps major white matter tract. Lesions of the forceps major, which connect bilateral occipital cortices, lead to topographical disorientation in humans (*73*). In addition, the *AUTS2* and *SH2B3* dyslexia-disposing variants were associated with higher volume in the optic radiation. Our findings may relate causally to visuo-orthographic deficits in dyslexia (*74*), or might signify a secondary adaptation of the visual network to lower reading activity in adults with higher genetic disposition (for example, due to reading avoidance). After adjusting for head size, we also saw increased volume of primary visual cortex in those with higher polygenic disposition to dyslexia, which may be consistent with these scenarios. However, as noted in Results, caution should be used when adjusting for heritable traits (such as head size) as confounding variables in genetic association analysis (*44*). As higher dyslexia PGS was associated with widespread volume reductions that affect a substantial proportion of the brain, then some of the “spared” regions, i.e., those that were not significantly affected in either direction, may appear to “increase” in volume after applying a global metric such as head size as a confound variable. From a statistical perspective, this issue parallels the long-debated practice of global signal regression in functional neuroimaging, which can sometimes cause spurious anticorrelation signals (*75*).

The association effect sizes between PGS and neuroimaging measures in our study were in the range of 0.2 to 1% of variance explained (and the impact modes based on independent component analysis explained totals of 25.5 and 8.5% of variance in brain-wide *t* maps for dyslexia-disposing variants, respectively in T1-weighted and dMRI data). This range of effect sizes is expected for polygenic effects based on common genetic variants, given that individual differences in complex behavioral traits in the general population typically show only subtle associations with neuroimaging measures, often requiring sample sizes of thousands of individuals for robust statistical detection and quantification (*26*). The value of the very subtle associations reported in the present study are not in terms of predictive biomarkers, but rather in terms of mapping specific brain regions and networks that associate with genetic disposition. The resulting brain maps based on structural and diffusion imaging can already give clues about dyslexia manifestation in the brain by querying affected regions with respect to their known functions. Furthermore, affected brain regions in terms of regional volume or AFD should be investigated in future studies at other, deeper levels of organization, such as spatial gene expression, neurotransmitter receptor density, or cytoarchitectonics, where effects of genetic disposition may be more apparent.

Our study has some limitations. Although the UK Biobank is a population sample, this cohort is healthier on average than the general UK population due to volunteering bias (*76*). Dyslexia PGS was derived using data from a large GWAS study of participants who self-reported having received a diagnosis of dyslexia (*30*), but with no information on the subtype, timing or severity of this condition (e.g., no distinction was made between acquired and developmental dyslexia, no information was recorded on who had made the diagnosis, and performance on dyslexia-related cognitive or behavioral tasks was not recorded). Nevertheless, strongly negative genetic correlations of this dyslexia phenotype with quantitative measures of word reading (*r*_g_ = −0.71) and spelling (r_g_ = −0.75) argue for its validity (*30*, *31*). While our “impact mode” analysis of polygenic disposition identified distinct sets of genomic factors associated with distinct brain regions, we do not claim to show in the present study that variation in these different brain regions is associated with different dyslexia-related cognitive traits. The present cross-sectional study was carried out using adult data, which means that cause-effect aspects are not possible to disentangle with certainty. Future large-scale imaging genetic studies would benefit from longitudinal data from children, to inform on genomic impact modes for brain changes before and during reading acquisition. There is evidence that the brain basis of dyslexia partly differs in boys and girls (*77*), but the polygenic dispositions that we calculated for UK Biobank participants were based on variant-wise effect sizes from a previous GWAS of dyslexia in which males and females were included together (*30*). Future work could examine possible sex differences in the neural correlates of genetic disposition to dyslexia in the UK Biobank, while calculating PGSs based on sex-specific GWAS results for dyslexia (*30*). Replication and/or cross-validation of our findings would be desirable, but we are unaware of another adult brain imaging genetics cohort of comparable scale.

In summary, we identified multiple brain networks linked to genetic disposition to dyslexia in a large adult sample from the general population. This approach complements classical case-control designs, for which it has not been possible to collect sample sizes within the same order of magnitude as that used here. Our study revealed that genetic disposition to dyslexia can be decomposed into distinct sets of genomic variants associated with distinct sets of brain regions. This decomposition is consistent with dyslexia as a complex and heterogeneous trait. Our study also showed which brain structural features are associated in common across multiple traits that are genetically correlated with dyslexia, as opposed to being associated with dyslexia genetic disposition alone among these traits.

## MATERIALS AND METHODS

### Experimental design

#### UK Biobank data

UK Biobank data were accessed following approval of the application number 16066, P.I. C.F. UK Biobank is an in-depth investigation of more than 500,000 volunteers in the UK who are assessed for health, lifestyle, genomic, and many other variables (*32*). Multimodal brain MRI data (*33*, *34*) had also been released for approximately 10% of the individuals when the present study was initiated in 2022 (*78*). The UK Biobank received ethical approval from the National Research Ethics Service Committee North West-Haydock (reference 11/NW/0382), and all of their procedures were performed in accordance with the World Medical Association guidelines. Written informed consent was provided by all of the enrolled participants.

Genotyping has been performed using either BiLEVE Axiom or Axiom arrays from Affymetrix, which target highly overlapping sets of ~800,000 genomic variants with more than 95% similarity (*79*). The UK Biobank has also released common genome-wide variants imputed to Haplotype Reference Consortium and UK10K haplotypes (*79*). In this study, we focused on participants who also underwent brain MRI at one of the four imaging sites and for whom at least one usable T1-weighted and/or dMRI scan had been produced (see the next section). The genetic analyses were focused to the largest ancestry group within this cohort, recorded as “white British” using a combination of self-report and genomic principal component analysis (this group constitutes ~85% of the overall dataset: data field #22006). Pairs of genetically related subjects with kinship coefficients above 0.044 were identified in the target sample (*70*). Individuals related to the largest number of others were recursively removed until no two individuals were related at or above this kinship threshold, leaving 35,231 individuals (18,363 females). The resulting sample encompassed individuals aged from 45 to 82 years, with a mean age of 64.2 years and an SD of 7.7 years. We then included bi-allelic genetic variants with minor allele frequency ≥ 0.01, imputation quality score of higher than 0.7, and Hardy-Weinberg equilibrium *P* value of greater than 10^−7^, yielding 8,366,177 autosomal single-nucleotide variants (SNVs) and 1,092,696 short insertion-deletions (indels).

We accessed minimally processed and brain-extracted T1-weighted brain MRI volumes of 42,798 individuals (*33*, *34*) for tensor-based morphometry using symmetric image normalization (SyN) registration (*37*, *38*). 

### Statistical analysis

#### Structural MRI: Tensor-based morphometry

For the present study, we generated a study-specific average brain template in a randomly chosen subset of 1000 individuals. The template was generated through 11 consecutive Advanced Normalization Tools (ANTs version 2.3.5) registrations that iteratively refined the template shape using rigid, affine, and diffeomorphic SyN transformations at incremental resolutions up to native resolution (i.e., 1 mm^3^). Thereafter, all individuals’ original T1-weighted brain volumes were histogram matched, winsorized at 1st to 99th percentiles, and nonlinearly registered to our study-specific template using SyN. Registration parameters included a variance for total field of three, and variance for update field of zero, a resolution downsampling scheme of 6×, 4×, 2×, and 1× (i.e., full resolution) and Gaussian smoothing at SDs of 4, 2, 1, and 0 voxels. A cross-correlation metric with a radius of four voxels was used.

The affine registration matrix was composed with the SyN deformation field and the final warps were subsequently converted to Jacobian determinant maps, which encode the amount of regional brain tissue “shrinkage” or “expansion” in the brain of each individual as compared to our study-specific, average T1 template. ANTs affine registrations failed in 2098 individuals; instead of removing them, we opted to use a comparable linear registration method, FSL Flirt (*80*) to initialize SyN, while controlling for a potential batch effect in subsequent analyses as a binary covariate.

#### dMRI: Data preprocessing and fixel-based analysis

We retrieved minimally preprocessed dMRI volumes of 37,930 subjects from UK Biobank (*33*, *34*). These data have been collected at 2 mm^3^ isotropic resolution across 100 different diffusion-encoding directions evenly distributed on two spherical shells at *b* values of 1000 and 2000 s/mm^2^, as well as eight blip-reversed *b* ≅ 0 volumes. Diffusion images have been corrected for off-resonance warps, gradient nonlinearity, Eddy currents, and head motion by the UK Biobank team (*33*, *34*). For the present study, we reran these corrections on raw data for a first batch of 8247 individuals whose corrected *b* vector tables were not available, while accounting for a potential batch effect in the subsequent regression model fits through the use of a binary covariate. After data preprocessing, we constructed a study-specific fiber orientation density (FOD) template using MRTrix3 version 3.0.3 (*81*) from a random subset of 890 individuals who passed registration quality control by visual inspection out of 1000. This procedure started by N4 bias field correction and intensity normalization of the preprocessed diffusion volumes, and estimation of the average tract response function (*82*). Thereafter, spherical deconvolution was performed using the estimated response function to generate subject-wide FOD volumes. These volumes were subsequently nonlinearly registered to a common space and an average FOD template was generated iteratively. The FOD template was then “fixelated” to identify the principal directions of white matter tracts in each voxel. The same procedures were repeated in all 37,930 individuals to generate FOD volumes, which were then registered to the study-specific FOD template (*81*). FOD registrations passed quality control in 37,884 individuals following visual inspection of each individual’s template-transformed zeroth-order harmonic map, representing average isotropic diffusion in each voxel. FOD volumes were segmented to obtain fixel-wise readouts, which were then transformed, rotated, and corresponded to the template’s fixel-wise space (*81*). We considered AFD readouts as a measure of white matter microstructure for subsequent analyses (*39*). In combination with genetic data, the sample available was 31,695 adult individuals (16,198 female).

#### Optimizing polygenic scoring

We first concatenated the voxel-wise Jacobian and fixel-wise AFD maps across all individuals and then applied MELODIC independent component analysis (*35*, *36*) to extract imaging-derived phenotypes (IDPs). MELODIC was performed separately per each imaging modality and at various dimensions to extract IDPs at incremental levels of spatial detail, following a geometric series corresponding with dimensions 11, 18, 29, 47, 76, 124, 200, and 324. Because of the large size of this data matrix (6.2 × 10^10^ voxels in structural MRI), we used 8000 internal eigenmaps for independent source decomposition (*83*). In addition, principal components analysis was performed on the same data and the first 324 principal components were extracted as additional IDPs. Together, a total of 1153 IDPs were extracted from voxel-wise Jacobian maps and an equal number of IDPs from the fixel-wise AFD data. These IDPs were derived for the purpose of optimizing our polygenic scoring, but they were not used for our voxel- or fixel-based imaging genetic analyses, nor our impact mode analysis, which form the bulk of the findings in this study.

We used summary statistics from the largest GWAS of dyslexia that has been performed to date, carried out by 23andMe Inc. (*30*). This GWAS was based on 51,800 individuals of European ancestry who answered “yes” to the question “have you been diagnosed with dyslexia?,” and 1,087,070 control individuals who answered “no.” The SNP-wise effect sizes from this GWAS were then applied to the genotype data of UK Biobank individuals, to estimate the polygenic disposition of each UK Biobank individual to dyslexia based on the combined effects of their autosome-wide genetic variants.

Our primary approach for polygenic scoring was based on the Lassosum2 model (*40*). We observed strong correlation between Lassosum2 PGS and two automated PGS methods, SBayesR (*41*) and PRS-CS_auto_ (*42*). Lassosum2 generally explained the highest proportion of variance in brain IDPs (fig. S1) and was therefore used for the main analysis. This method fits a sparse elastic-net regression and optimizes two shrinkage penalties, including L1-norm (λ) and L2-norm (δ). A grid search across 30 λ and 10 δ values was used for optimization with respect to maximizing the top association with any IDP. The associations of dyslexia PGS were quantified with all 1153 IDPs in each imaging modality using linear regression. A set of confound covariates were controlled for, including subject age at imaging visit (data field #21003, instance 2), age^2^, sex (data field #31), age × sex, age^2^ × sex, the first 10 principal components of genomic ancestry (data field #22009), genotyping array (data field #22000, either BiLEVE or Axiom), three dummy covariates encoding four UK Biobank neuroimaging sites (data field #54, instance 2), and the number of days passed since MRI scan incepted at the site (as a measure of slow drifts in MRI hardware performance; data field #53, instance 2). For structural MRI data, the type of affine registration (i.e., ANTs or Flirt) was further controlled as a covariate. Structural MRI analysis was performed either without (main analysis) or with (secondary analysis) correction for head size as a confounding covariate (data field #25000). For dMRI data, the batch effect associated with diffusion preprocessing (i.e., either performed by our team or by the UK Biobank) was added to the covariates, and the analyses were done without (main analysis) and with (secondary analysis) the global mean apparent fiber density per individual as an extra covariate. We found that high δ values in the range of 10^2^ to 10^4^ slightly increased the accuracy of Lassosum2 over automated models PRS-CS_auto_ and SBayesR, and λ in the range of 10^−5^ to 10^−2^ resulted in the highest accuracy of trait prediction (fig. S1). These shrinkage parameters were therefore used for subsequent analyses.

#### Voxel- and fixel-wise brain associations with dyslexia PGSs

We tested the brain-wide associations of dyslexia PGS with the voxel-wise and fixel-wise data in the UK Biobank. Both parametric [fsl_glm 6.0.3 (*84*)] and nonparametric [randomise version 2.9 (*85*)] linear regression models were fitted to the data, the former to yield *t* value maps for visualization and impact mode analysis, and the latter to generate brain-wide multiple-comparisons–corrected *P* value maps. To reduce computation costs, voxel-wise permutations were performed at half (2-mm^3^ isotropic) resolution with a wall time of 9 days for 5000 permutations per statistical contrast. The Randomise C++ code was modified to prevent short integer overflows due to the study sample size. No cluster enhancement was applied. The same sets of covariates as the previous section were used as for optimization. In all cases, we observed that a parametric *t* value of >4.5 was equivalent to a nonparametric brain-wide–corrected *P* value of smaller than 0.05.

As a check on the validity of our findings obtained with Lassosum2, we applied other methods for deriving PGS: SBayesR, PRS-CS, and PRS-CS_auto_. PRS-CS applies continuous shrinkage on variant-wise weights using Bayesian priors and is optimized using a single global shrinkage hyperparameter (ϕ). We explored four different ϕ values for optimizing PRS-CS, which were 10^−6^, 10^−4^, 0.01, and 1 (fig. S1). PRS-CS_auto_ and SBayesR are automated polygenic scoring methods and therefore did not require hyperparameter optimization on an independent dataset. We found that dyslexia Lassosum2 PGS was strongly correlated with dyslexia PGS derived from PRS-CS_auto_ (Pearson’s *r* = 0.87 and 0.93 following optimization on structural or diffusion-derived measures, respectively) and SBayesR (*r* = 0.74 and 0.84, same order). Compared to Lassosum2, these additional PGS exhibited highly similar brain-wide associations (fig. S2). To describe the white matter tracts that run through regions where fixels showed significant associations of AFD with dyslexia PGS, we ran probabilistic fiber tractography using the second-order Integration over Fiber Orientation Distributions (iFOD2) algorithm in the template space (*86*).

#### Dyslexia locus-based neuroimaging association

Forty-two individual genomic loci were significantly associated with dyslexia after genome-wide multiple testing correction in the 23andMe Inc. GWAS for dyslexia (*30*). Thirty-five of these variants passed our genetic quality control process in the UK Biobank data (see the “UK Biobank data” section above). At each of these 35 loci, dosage of the dyslexia disposing allele was calculated and used in separate linear regression models to find brain-wide associations with regional volume and white matter microstructure (i.e., voxel-wise Jacobian values and fixel-wise AFD values, respectively), using the same approach and covariates as when testing voxel-wise and fixel-wise PGS associations. These covariates included age, age^2^, sex, age × sex, age^2^ × sex, 10 principal components of genomic ancestry, genotyping array, UK Biobank imaging site, the number of days passed since MRI scan incepted at the site, the type of affine registration (for structural MRI), and preprocessing being either performed by our team or by the UK Biobank team (for dMRI). For each variant, we also performed secondary analyses in which head size or subject-average AFD across all fixels were additionally included as confound covariates, respectively in T1 and diffusion data modalities.

#### Impact mode decomposition

PGSs approximate polygenic influences through a single scalar value. These models represent a weighted average of all disposing allele counts and are agnostic to variability in the brain-wide associations of genetic variants. We aimed to model the heterogeneity and the hidden covariance patterns in the brain-wide genomic associations. To achieve this, we initially created a brain-wide univariate association map (i.e., voxel-wise or fixel-wise *t* value maps generated by a parametric regression) for each of the top independent 13,766 dyslexia GWAS loci, after clumping at a GWAS *P* value threshold of less than 0.01, linkage disequilibrium *r*^2^ threshold of less than 0.1 and genomic window size of 500 kb (and using the same set of covariates as in all sections above). These voxel- or fixel-wise *t* value maps were then concatenated across all 13,766 variants and decomposed by MELODIC into 10 independent components, separately per imaging modality. To enhance the sensitivity of ICA rotations to local effects rather than genetic associations with global measures, voxel-wise Jacobian determinant values were normalized to total brain volume before ICA. The default MELODIC ICA data transformations, including variance normalization and mean signal removal, were not applied as these momentums reflect meaningful signals in *t* value maps (*87*). We refer to the extracted independent components as genomic impact modes, which reflect combinations of distinct genomic variants and spatial profiles through a limited number of features.

#### PGSs of additional traits related to dyslexia

We first used LD score regression (*88*, *89*) to confirm that we could detect previously reported genetic correlations between dyslexia and each of eight other behavioral, cognitive, or education-related traits, based on summary statistics from the 23andMe dyslexia GWAS (*30*) and other large-scale GWAS studies: ADHD (*90*), verbal numerical reasoning (also known as fluid intelligence) (Pan-UKB team, https://pan.ukbb.broadinstitute.org), the first principal components of school grades in mathematics and language (*91*), General Certificate of Secondary Education (GCSE) education (Pan-UKB team, https://pan.ukbb.broadinstitute.org), word reading, nonword reading, spelling, and phonemic awareness (*31*). All of these traits showed significant genetic correlations |*r*_g_| > 0.4 with dyslexia in our analysis (all *P* < 10^−23^; fig. S4).

To compare and contrast with dyslexia PGS, we then used Lassosum2 to generate PGS in the UK Biobank data for each of these eight additional traits, and mapped their brain-wide associations with the voxel-wise and fixel-wise data, using the same approach as for the dyslexia PGS.

#### Further post hoc regression analyses

We performed further regression analyses of the association between dyslexia PGS and voxel-wise volumes, this time using logarithm-transformed Jacobian determinant values to take allometry into account (*92*), rather than raw values, to assess whether this made a difference. In another post hoc analysis, two extra covariates were added to assess voxel-wise and fixel-wise associations with dyslexia PGS independently of fluid intelligence and educational attainment: These covariates were “fluid intelligence” (data field #20016) and the number of years of education estimated from the data fields “qualifications” (#6138) and “age completed full-time education” (#845), following a previously published approach (*93*). Apart from the inclusion of these two covariates, the linear regression models were the same as the primary analyses described above in the section “Voxel- and fixel-wise brain associations with dyslexia PGSs.”
