# Supplementary material for: Distinct impact modes of polygenic disposition to dyslexia in the adult brain
Source: Sci Adv. 2024 Dec 18;10(51):eadq2754. doi: 10.1126/sciadv.adq2754 (PMC11654687; doi:10.1126/sciadv.adq2754)
Supplement: Supplementary file 1 — Figs. S1 to S6 Legends for tables S1 and S2 Legend for data S1 [file sciadv.adq2754_sm.pdf]

Science Advances
AAAS

Supplementary Materials for

Distinct impact modes of polygenic disposition to dyslexia in the adult brain

Sourena Soheili-Nezhad *et al.*

Corresponding author: Clyde Francks, clyde.francks@mpi.nl

Sci. Adv. **10**, eadq2754 (2024)
DOI: 10.1126/sciadv.adq2754

The PDF file includes:

Figs. S1 to S6
Legends for tables S1 and S2
Legend for data S1

Other Supplementary Material for this manuscript includes the following:

Tables S1 and S2

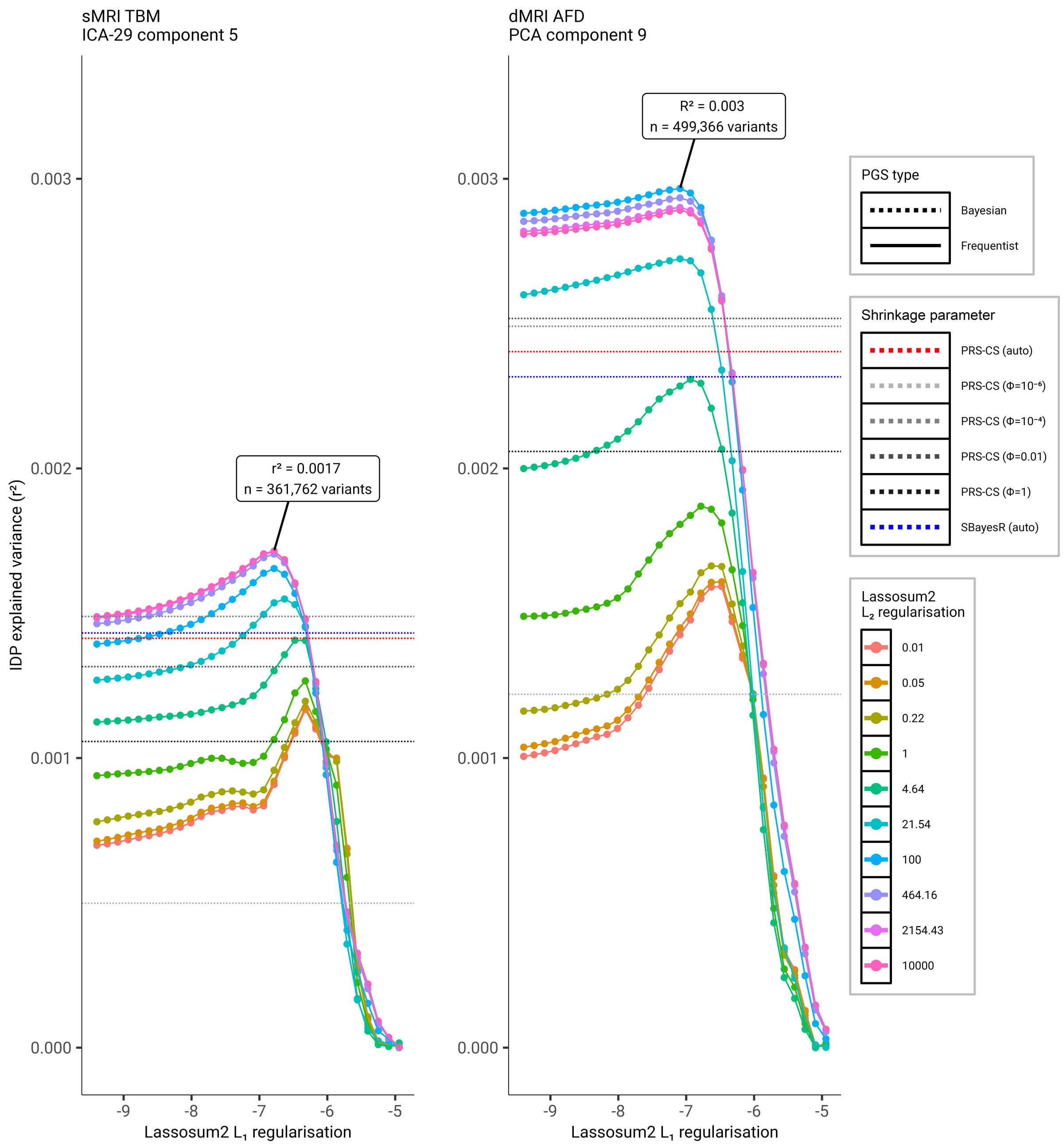

Fig. S1. Optimization process for dyslexia polygenic score models using phenotypes derived from tensor-based morphometry (left) and fixel-based analysis (right). The Lassosum2 polygenic scores were optimized by adjusting the L1 and L2 regularization parameters. Additionally, we compare the results of two automated polygenic scoring methods, SBayesR and PRS-CS auto, represented as dashed blue and red lines, respectively. Furthermore, we explore the manual optimization of PRS-CS scores (dashed grey lines), employing four different shrinkage parameters. IDP: imaging-derived phenotype. AFD: apparent fiber density. TBM: tensor-based morphometry. PGS: polygenic score.

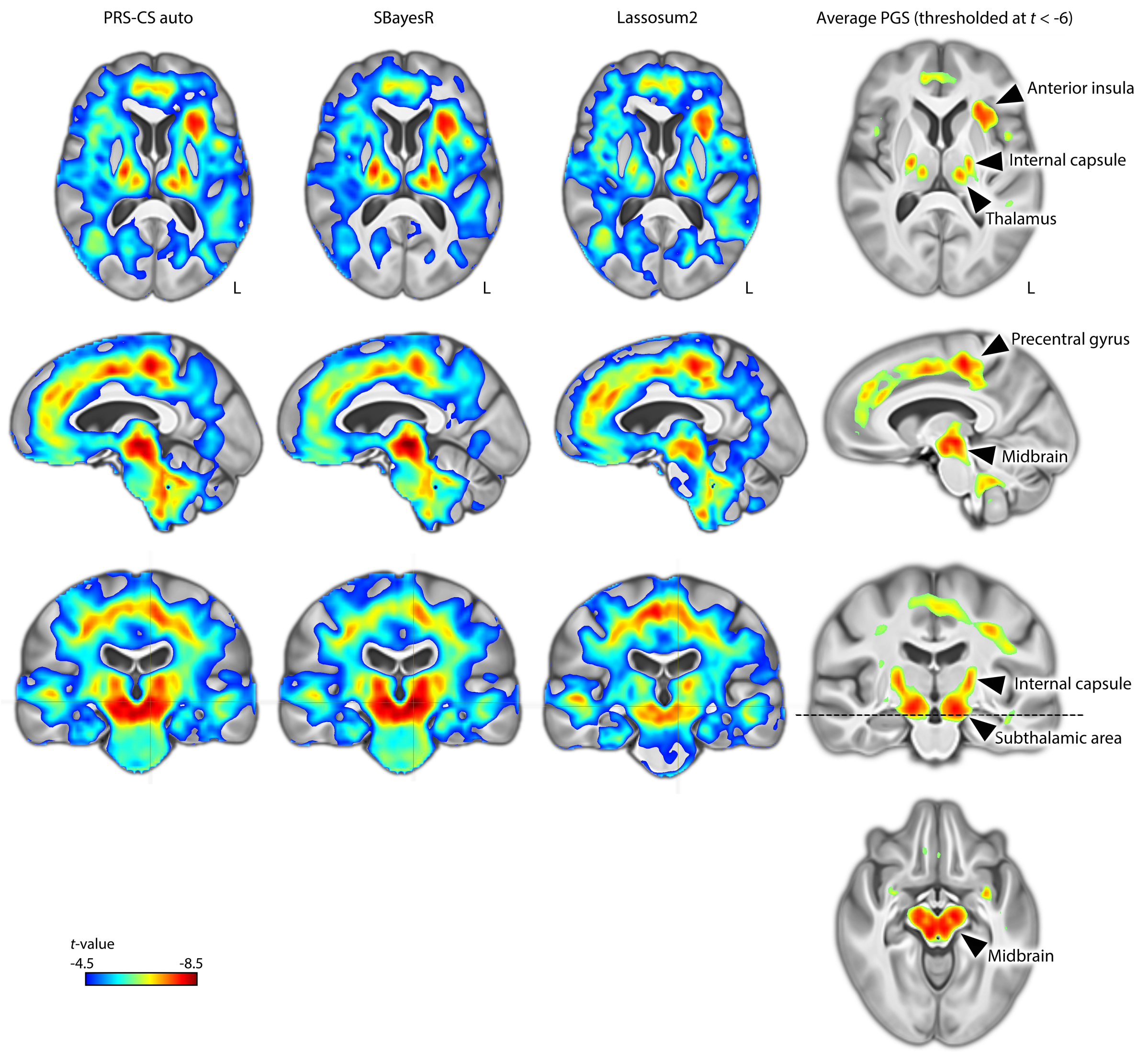

Fig. S2. Dyslexia polygenic scores generated by three different polygenic methods and their voxel-wise associations with regional brain volume. Triangles represent the coordinates of statistical peaks in an average statistical map of all three polygenic models.

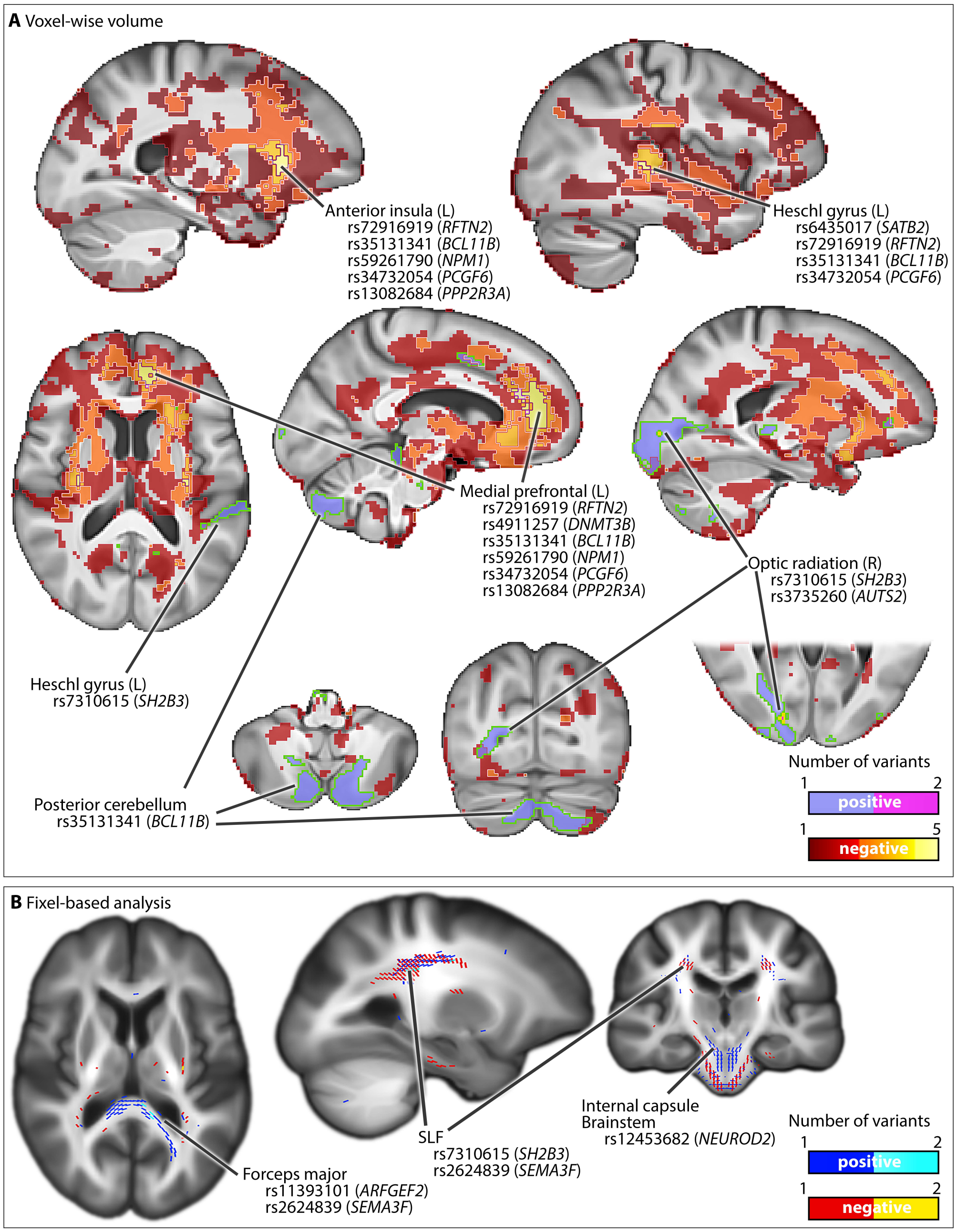

Fig. S3. Brain-wise volumetric (top) and fixel-wise apparent fiber density (bottom) association maps in the UK Biobank data, for 35 genetic variants that were significantly associated with dyslexia at a genome-wide significant level in the 23andMe GWAS. Values indicate the number of variants at each voxel or fixel that show significant positive or negative association with respect to the dyslexia-disposing allele, as obtained from a non-parametric test following 5000 permutations. Note that the overlap in terms of affected brain regions is generally low among these dyslexia-associated variants, as the few regions of overlap involve no more than 6 of the 35 variants. Separate maps for all 35 variants are provided in Data S1. SLF: superior longitudinal fasciculus.

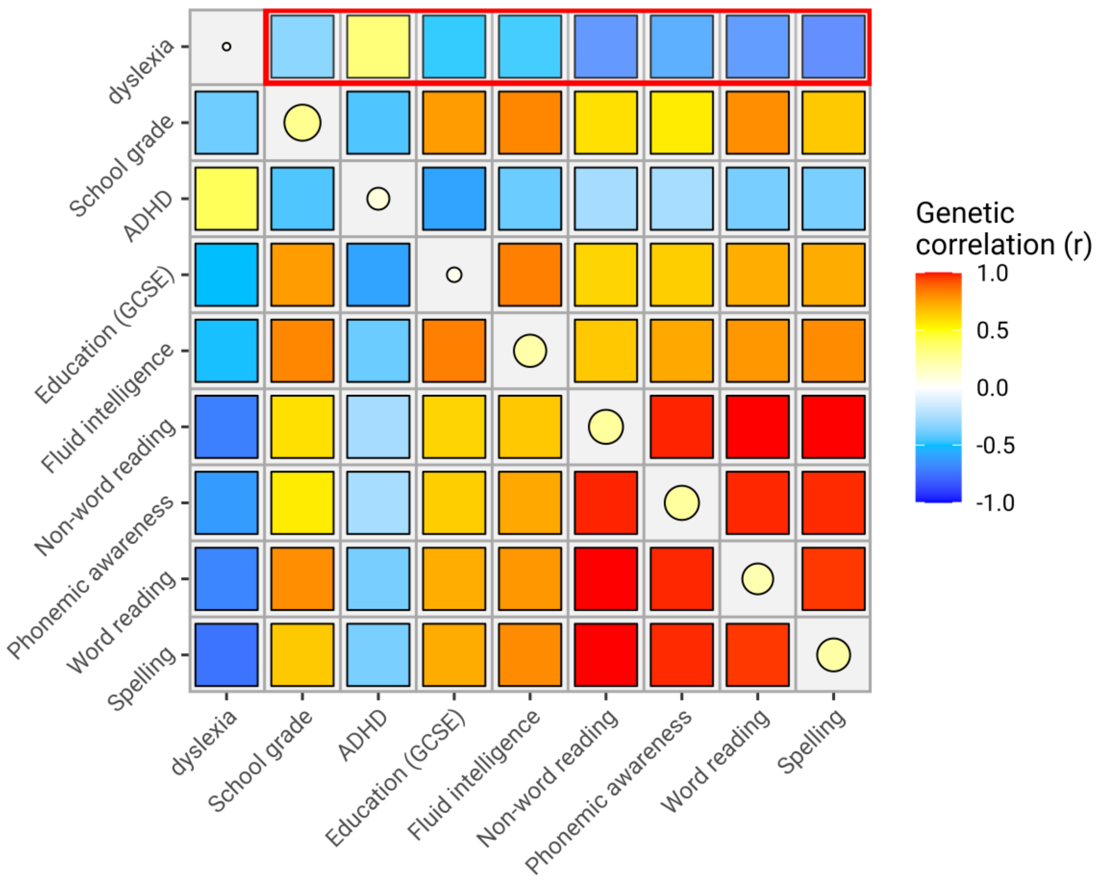

Fig. S4. Genetic correlations of dyslexia with other traits (see Methods for the data sources).

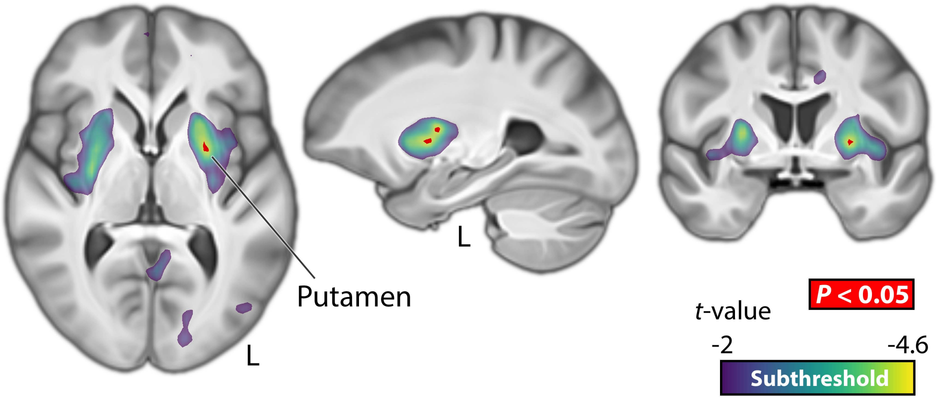

Fig. S5. Brain-wide association of spelling performance polygenic scores. Higher PGS for better spelling performance is associated with lower putamen volume. t-values indicate parametric regression tests. Voxels passing brain-wide multiple comparisons correction following 5,000 permutations are shown in red.

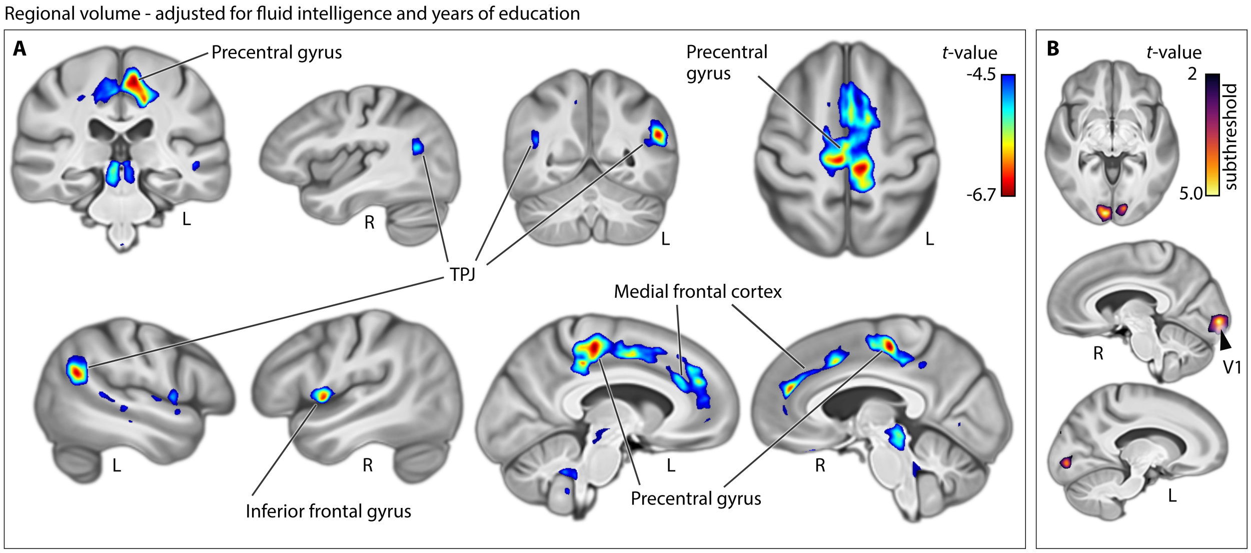

Fig. S6. Associations of dyslexia polygenic scores (PGS) with regional brain volume after adjustment for fluid intelligence and years of education as additional covariates. The rest of the covariates were the same as in the primary analysis (see Materials and Methods). In panel A the rainbow clusters indicate regions whose volumes were significantly lower in individuals with higher polygenic disposition to dyslexia, at p-values of smaller than 0.05 as obtained from non-parametric testing, with brain-wide correction for multiple comparisons using 5000 permutations. In panel B, regions whose volumes were higher in individuals with higher polygenic disposition to dyslexia are indicated. Most of these were subthreshold (i.e. non-significant) and only a small number of voxels in the primary visual cortex passed significance (arrowhead). Figures are shown in radiological convention, where the left side in transverse and coronal views corresponds to the right cerebral hemisphere and vice versa. R: right. L: left. TPJ: temporo-parietal junction.

Table S1. (separate Excel file)
Variant-wise impact mode weights for tensor-based morphometry ICA

Table S2. (separate Excel file)
Variant-wise impact mode weights for apparent fiber density ICA

Data S1. (deposited on Dryad: https://doi.org/10.5061/dryad.80gb5mkz6)
Analysis scripts and data files of the 3D brain maps corresponding to the visual items presented in this manuscript
